# Role of Epileptic Activity in Older Adults With Delirium, a Prospective Continuous EEG Study

**DOI:** 10.3389/fneur.2019.00263

**Published:** 2019-03-19

**Authors:** Sara Sambin, Nicolas Gaspard, Benjamin Legros, Chantal Depondt, Sandra De Breucker, Gilles Naeije

**Affiliations:** ^1^Neurology Department, ULB-Hôpital Erasme, Université Libre de Bruxelles (ULB), Brussels, Belgium; ^2^Geriatrics Department, ULB-Hôpital Erasme, Université Libre de Bruxelles (ULB), Brussels, Belgium

**Keywords:** delirium, NCSE, interictal discharges, continuous EEG, seizures

## Abstract

**Background/Objectives:** Delirium occurs in up to 50 % of hospitalized old patients and is associated with increased morbidity and mortality. Acute medical conditions favor delirium, but the pathophysiology is unclear. Preliminary evidence from retrospective and prospective studies suggests that a substantial minority of old patients with unexplained delirium have non-convulsive seizures or status epilepticus (NCSE). Yet, seeking epileptic activity only in unexplained cases of delirium might result in misinterpretation of its actual prevalence. We aimed to systematically investigate the role of epileptic activity in all older patients with delirium regardless of the underlying etiology.

**Design, Setting:** Prospective observational study in a tertiary medical center. Adults >65 years with delirium underwent at least 24 h of continuous electro-encephalographic monitoring (cEEG). Background patterns and ictal and interictal epileptic discharges were identified, as well as clinical and biological characteristics.

**Participants:** Fifty patients were included in the study.

**Results:** NCSE was found in 6 (12%) patients and interictal discharges in 15 (30%). There was no difference in the prevalence of epileptic activity rates between delirium associated with an acute medical condition and delirium of unknown etiology.

**Conclusion:** Epileptic activity may play a substantial role in the pathophysiology of delirium by altering brain functioning and neuronal metabolism. No clinical or biological marker was found to distinguish delirious patients with or without epileptic activity, underlining the importance of cEEG in this context.

## Introduction

Delirium occurs in up to 50% of hospitalized older patients (1, 2) and is defined as an acute, transient, and potentially reversible alteration of cognitive function. Patients with delirium are twice as likely to die or to be institutionalized at 24 months and ten times more likely to develop incident dementia (1, 3).

Delirium complicates a wide range of acute medical conditions such as, e.g., infections, electrolytic imbalances, post-surgical state… The unifying mechanism underlying the development of delirium in these conditions is still unknown (4–6). A multifactorial model proposes that delirium, represents the clinical manifestation of an “acute brain failure,” resulting from a diminished capacity of a previously dysfunctional brain (e.g., reduced brain connectivity and brain plasticity) to compensate for an acute stressful event (2, 5), similarly to acute exacerbation of chronic renal failure. Although such model sets a good framework for the understanding of delirium, it does not provide a pathophysiological mechanisms and therefore confines the therapeutic interventions to symptomatic treatment.

A growing body of evidence suggests that a proportion of older patients with delirium may have non-convulsive status epilepticus (NCSE) (7–14). Seizures and status epilepticus induced by precipitating factors (electrolytic imbalances, alcohol withdrawal, drug intoxication…) could represent one of the missing links between acute medical stressful events and delirium. Indeed, both acute symptomatic seizures and delirium occur more often in older people and share common predisposing and precipitating factors (5, 7, 8, 13, 15).

A pilot study disclosed that 24 h continuous electroencephalographic monitoring (cEEG) detected ictal (NCSE) and epileptic discharges in 14/32 (44%) of older adults who presented with delirium, and that these ED occurred more frequently in subjects with infection, higher blood urea nitrogen/ creatinine ratio (BUN/Creat) and cognitive impairment (9). Those results suggest that epileptic discharges may play a role in delirium pathophysiology and highlight the yield of cEEG in that context. However, this study compared routine EEG and cEEG in a limited number of patients. So, the high percentage of epileptic discharges in the cEEG group might be the consequence of a selection bias, with more severe, and unexplained cases undergoing cEEG rather than routine EEG, and therefore potentially overestimating the proportion of NCSE and epileptic discharges.

In this work, we aimed to confirm and clarify those findings in a prospective and larger cohort of older adults with new onset delirium, irrespective of etiology, and studied exclusively with cEEG in order to minimize selection bias. We also aimed to identify the risk factors of epileptic patterns.

## Methods

### Design

This was a prospective study from December 2016 to September 2017 performed at Cliniques Universitaires de Bruxelles—Hôpital Erasme, Brussels, Belgium. We included patients >65 years old with delirium onset <24 h. Patients were recruited from the emergency, neurology, and geriatrics departments by the main author (SS) during week days upon contact with the physicians in charge of the patient who identified the presence of a delirium using the Confusion Assessment Method (CAM) criteria for delirium (16). Identified cases were reviewed by the primary author (SS). Patients presenting with delirium following witnessed tonic-clonic seizures were excluded.

### EEG

All included patients underwent cEEG for at least 24 h.

cEEGs were recorded using 21 scalp electrodes placed according to the international 10–20 system. They were reviewed at least twice daily by neurologists trained in cEEG (NG, BL, CD). Periodic, rhythmic, and sporadic epileptiform discharges were classified according to published consensus criteria (17–19) and classified into: Generalized periodic discharges (GPDs), bilateral independent periodic discharges (BIPDs), lateralized periodic discharges (LPDs) and focal, generalized, bilateral independent, and multifocal sporadic epileptiform discharges (SEDs). Non-convulsive seizures (NCSz) and NCSE were defined according to the consensus criteria (20).

The anti-seizure treatment was managed at the discretion of the treating physician and individualized according to the type of SE/seizure, co-morbidity, and co-medications. Our institutional protocol for the treatment of NCSE was previously described (21).

### Clinical Data

Demographic, clinical, biological, and imaging data, length and outcome of hospitalization were extracted from the medical records. Focal neurological symptoms and signs, as well as vital parameters were recorded on the day of admission and day of inclusion. Main risks factors for delirium from validated predictive models and medical causes of seizures were used for comparisons between group with and without PDs/NCSE/NCSZ (1, 5, 22). Specifically, medical history for stroke, cognitive disorders and epilepsy, were sought as well as use of psychoactive drugs. Results from cerebral imaging performed with CT scan, MR, or both for each patient was retrieved. Hemoglobin concentration, blood urea nitrogen/ creatinine ratio (BUN/Creat), and CRP were chosen for comparisons for their known frequent association with delirium occurrence and prognostic (23–26) as well for the trends identified in the pilot study (27). The etiologies of delirium were also reported.

### Statistics

Results are presented as mean (±SD), median (range) or count (percentages) as appropriate.

Comparisons between the proportions of patients with periodic discharges or NCSE with patients with sporadic or no discharges were performed with respect to qualitative and quantitative variables. History of cognitive impairment, stroke and epilepsy, as well as the percentage of patients with chronic and acute brain lesions, delirium type, and fatal outcome were compared between groups by Fisher exact test. Mann-Whitney test was performed to compare numeric variables in the two groups.

### Data Sharing Statement

Extra data are available upon request by emailing the corresponding author.

## Results

### Study Cohort

Fifty patients were included between December 2016 and September 2017. Mean age was 84 ± 6 years, and 33 (66%) were females. Delirium was hyperactive in 7 subjects of whom 2 presented acute psychoactive drugs withdrawal and 1 had ethylic dementia. Underlying delirium precipitating factor could be identified in 35 patients (70%) and included: 17 infections (34%), 4 infections and dehydrations and/or acute renal failure (8%), 3 dehydrations and/or metabolic imbalances (6%), 6 post-surgery states (12%), 2 urinary retention/fecal impactions (4%), 3 drug/alcohol intoxications or withdrawals (6%). Cerebrospinal fluid was analyzed in eleven patients and within normal limits (Mean value ± SD: protein concentration 40.7 ± 19 mg/dl, normal values 15–45 mg/dl; cell count 1.8 ± 1.6, normal values >5 and glycorachia 99.77 ± 48.5 mg/dl, normal values 45–80 mg/dl). Other main characteristics and clinical features of the study cohort are summarized in Table 1.

**Table 1:** Cohort characteristics.

	**N (%) mean (SD) or median (IQR)**
**Age (years)**	84 ± 6
**Women (*****n*****, %)**	33 (66%)
**History of stroke (*****n*****, %)**	18 (36%)
Ischemic	14 (28%)
Hemorrhagic	4 (10%)
**History of epilepsy (*****n*****, %)**	3 (6%)
**Cognitive impairment (*****n*****, %)**	17 (34%)
Degenerative	4 (8%)
Vascular dementia	6 (12%)
Mixed dementia	2 (4%)
Unspecified	5 (10%)
**Psychoactive drugs (*****n*****, %)**	25 (50%)
**DELIRIUM TYPE (*****n*****, %)**	
Hypoactive	38 (76%)
Hyperactive	7 (14%)
Mixed or undefined	5 (10 %)
**BIOLOGICAL VALUES (MEAN ± SD)**	
Hb (mg/dL, normal range: 13–16)	12.2 ± 3.0
Urea/creatinine ratio	50 ± 25
CRP (mg/L, normal range < 10)	64.9 ± 76.6
**IMAGING (*****n*****, %)**	
Acute brain lesion	12 (24%)
Ischemic stroke	6 (12%)
Hemorrhagic stroke	6 (12%)
Chronic focal lesion	10 (20%)
Microangiopathy	13 (26%)
Cerebral atrophy	2 (4%)
Microangiopathy and atrophy	7 (14%)
**Length of stay (days)**	18 (13–25)
**Death (*****n*****, %)**	11 (22%)

### EEG

#### Background Activity

Forty-nine patients had background EEG pattern anomalies, mild generalized slowing being the most frequent abnormal finding (see details in Table 2).

**Table 2:** EEG characteristics.

	***n* (%)**
**EEG BACKGROUND**	
Mild generalized slowing	40 (80%)
Moderate generalized slowing	8 (16%)
Severe generalized slowing	1 (2%)
Focal slowing	18 (36%)
Focal attenuation	10 (20%)
**Sporadic discharges**	10 (20%)
Focal	6 (12%)
Multifocal	3 (6%)
Bilateral independent	1 (2%)
**Periodic discharges**	11 (22%)
GPDs	8 (16%)
LPDs	3 (6%)
**Seizures**	7 (14%)
NCSE	6 (12%)

#### Sporadic Epileptiform and Periodic Discharges

Sporadic epileptiform discharges (SEDs) were detected in 10/50 patients (20%) and were focal or multifocal in 9/10 patients. Periodic discharges (PDs) were observed in 11/50 patients (22%) and were generalized in 8/11 patients.

Seven patients (14%) had association of sporadic and periodic discharges; 2 (4%) had focal sporadic discharges and LPDs and 5 (10%) had multifocal sporadic discharges and GPDs.

#### Seizures

Seizures or SE occurred in 7/50 (14%) patients. One patient had discrete right frontal lobe NCSz and six met the criteria for NCSE (three met frequency or evolution criteria, one had subtle myoclonus and two showed clinical and EEG improvement upon treatment). Four of the six NCSE were generalized and two were hemispheric at the time of the EEG.

Two of the six patients with NCSE had acute ischemic stroke and the patient with focal seizures had a subdural hematoma. One patient with NCSE had alcohol withdrawal.

#### Response to Antiepileptic Drug (AED) Treatment and Outcome

Twenty patients were treated with AEDs (40%), four by modification of a preexisting AED treatment that they were taking for past history of seizures (*n* = 2) and headache prevention (*n* = 1). Sixteen out of twenty patients had AEDs administered intra-veinously. The first line of AED administered was LEV levetiracetam (LEV) in 15/50, valproate (VPA) in 3/50, benzodiazepines (BZD) in 1/50, and lamotrigine in 1/50. Six patients needed the addition of second line AEDs (LEV in 3, VPA in 1, BDZ in 1, pregabaline in 1).

Of 20 patients treated with AEDs, six patients received the drugs before undergoing cEEG for clinical suspicion of NCSE and fourteen after the cEEG was started. In the patients treated before the onset of cEEG recordings, cEEG performed after AED treatment revealed SEDs in two and no PDs/NCSE/NCSz in 4; among them, four improved clinically (including one with SEDs). Patients having treatment under cEEG received AEDs for NCSE/NCSz in seven cases, PDS in four cases, MF, and bilateral independent discharges in, respectively, two and one patients. All patients with NCSE and NCSz showed clinical and EEG improvement, 3 patients with PDs out of 4 showed EEG improvement and 2 out of 4 showed clinical improvement, one patient with MF improved clinically and the patient with bilateral independent discharges displayed only EEG improvement.

Eleven patients (22%) died during hospitalization, due to underlying medical conditions or neurological complications: sepsis/infection in four cases, infection and stroke in two cases, infection and heart failure in one case, heart failure in two cases, renal failure and MF in one case and multiple causes in one. All the patients with NCSE were discharged from the hospital. Sixteen patients (32%) returned to their home; the others were transferred to rehabilitation units (*n* = 10.20%) or nursing facilities (*n* = 13.26%). Of the 20 patients treated, 6 died, 5 returned home, 2 were transferred to nursing facilities, and 7 to rehabilitation structures.

#### Delirium Precipitating Factors

Underlying delirium precipitating factor could be identified in 35 patients (70%) and included: 17 infections (34%), 4 infections and dehydrations and/or acute renal failure (8%), 3 dehydrations and/or metabolic imbalances (6%), 6 post-surgery states (12%), 2 urinary retention/fecal impactions (4%), 3 drug/alcohol intoxications or withdrawals (6%). Most of the patients with SEDs (7/10) and PDs (9/11) as well as two thirds of patients without epileptic activities (19/29) had at least one possible precipitating factor for delirium.

#### Biomarkers in Patients With or Without PDs/NCSE/NCSz (Table 3)

The proportion of women was significantly higher in the PDs/NCSE/NCSz group (*p* < 0.05). Otherwise, there was no difference for history of stroke (*p* = 0.5), history of epilepsy (*p* > 0.99), history of cognitive impairment or dementia (*p* = 0.73), nor delirium type (*p* > 0.99). The percentage of chronic and acute brain lesions detected by imaging was comparable in the two groups (*p* = 0.23 and *p* = 0.15, respectively). There was no difference of mortality (*p* = 0.23).

**Table 3:** Markers in patients with or without PDs/NCSE/NCSz.

	**PDs/NCSE/NCSz (*N* = 11)**	**No PDs/NCSE/NCSz (*N* = 39)**	***p***
Females (*n*, %)	10 (91%)	23 (59%)	0.07
Age (years, mean +/SD)	88.28 ± 6.49	83.23 ± 7	0.27
History of stroke (*n*, %)	3 (27%)	16 (41%)	0.5
History of epilepsy (*n*, %)	0	3(8%)	>0.99
Cognitive impairment/Dementia	3 (27%)	14 (36%)	0.73
Psychoactive substance use (*n*, %)	5 (45%)	16 (41%)	>0.99
**DELIRIUM TYPE (*****n*****, %)**			
Hypoactive	9 (82%)	29 (74%)	>0.99
Hyperactive	1 (9%)	6 (15%)	>0.99
Mixed	1 (9%)	4 (10%)	>0.99
**IMAGING**			
Acute brain lesion	4 (36%)	12 (31%)	0.73
Chronic focal brain lesion	0	10 (25%)	0.15
**BIOLOGICAL VALUES**			
Hb (mg/dL, normal range 13–16)	13.50 ± 4.7	12.20 ± 2.05	0.66
Urea/creatinine ratio	40.00 ± 24.41	44.83 ± 29.56	0.27
CRP (mg/L, normal range < 10)	23.50 ± 55.25	41.50 ± 80.75	0.82
Death (*n*, %)	4 (36%)	16 (18%)	0.23
Length of stay (days)	28.00 ± 13	16.50 ± 10.25	0.14

No biological anomalies were significantly associated with PDs/NCSE/NCSz (*n* = 11) compared to with patients with SEDs or no discharges (*n* = 39). e.g., no significant difference between were found for hemoglobin concentrations (*p* = 0.66), BUN/Creat (*p* = 0.27) and CRP concentration (*p* = 0.82). There was no significant difference either for patients' age (*p* = 0.27) or length of hospital stay (*p* = 0.14).

## Discussion

In this prospective cEEG study of older adults with delirium of unselected etiology, we found epileptic discharges in 42% of cases and electroencephalographic seizures in 14 % of cases, suggesting a potential role of epileptic activities in delirium pathophysiology.

Our study sample of hospitalized older adults matches the characteristics of previous studies on older patients with delirium in terms of age, sex, delirium type, psychoactive drug use, history of dementia or cognitive impairment (5, 28–31) and displays a similar proportion of past stroke and seizure history compared to previous studies investigating delirium associated with epileptic activities (8, 13). The referral situation of our center could bias toward more neurological cases and lead to increased prevalence of epileptic activities. However, acute brain injuries are regularly co-occurring in older adults' delirium studies amounting to 30% of cases (5, 32) and, in our cohort, acute brain injuries are not more prevalent in the group of patients with delirium and PDs/NCSE/NCSz. Furthermore, the proportion of infections, mean hemoglobin concentration, BUN/Creat and CRP levels in our patients matches the anomalies reported in older adults delirium studies (33–37) which suggests that our cohort reflects hospitalized older adults with delirium.

In analyzing cEEG, we found that generalized slowing was the rule (98%), with mild generalized slowing being the most frequent anomaly (80%), consistent with the known association of diffuse slowing with increased delta, and theta activity and delirium (26). In terms of ictal activities, compared to our pilot study (10), cEEG disclosed NCSE in fewer cases (12 vs. 28%). This findings is probably explained by the fact that NCSE is probably underestimated in this study, as six patients in our cohort (12%) received AEDs on clinical bases prior to the beginning of EEG (high clinical suspicion of NCSE,4 of them showed clinical improvement) and to the probable existence of an inclusion bias in our pilot study that allocated more often severe and unexplained cases more to cEEG. The proportion of NCSE in this study (12%) is in line with the 16% reported for NCSE in an EEG study that focused on delirium unexplained by biological or brain imaging abnormalities (8) while we included patients irrespectively of the delirium underlying etiology. However, although apparently selecting patients for whom an etiology for delirium had not been established, only one of the patients in that prior series ended as “cryptogenic” NCSE, the others had concomitant history of stroke, withdrawal of benzodiazepines, and/or meningo-encephalitis (8). These data underline the difficulty of distinguishing between “known” and “unknown” origins of delirium: causes of “unknown” delirium might simply be harder to detect on screening investigations. As in previous studies, no clinical, or para-clinical parameters could be singled out as a risk factor for epileptic activities in older adults with delirium (13), confirming the rationale for including patients with a known etiology. For instance, cEEG realized for altered mental status in the intensive care unit in patients with acute medical conditions (excluding primary neurologic diagnostics) detects 16% of non-convulsive seizures (38) suggesting that epileptic activities might play a role in delirium pathophysiology and reflect acute brain dysfunction whether being triggered by acute medical conditions or not (2, 5).

SEDs were detected in a third of subjects, which is comparable to our previous study. While the direct relation between ictal activity and clinical manifestations of delirium has been ascertained by several studies and confirmed by the present one (7, 8, 11–14, 27, 33), the role of interictal activities in delirium symptoms is unclear. Several indirect arguments suppose a link between interictal epileptiform activities, brain dysfunction, and delirium. First, the proportion of SEDs we report in older adults with delirium population is higher than in non-epileptic healthy adults (0.6–2.2%) (39) and even more so, if we consider that interictal epileptiform activity occurs less often in older epileptic patients (40). Second, SEDs are known to impair cognitive task performances, impacting working memory, and short-term memory related tasks, in close temporal relation with spiking activity which reverse after SEDs resolution (41–43). Third, epileptic activities are associated with nefarious metabolic changes in the brain (increase in oxygen and metabolite need for neurons, increased cerebral blood flow, raised intra-cranial pressure and higher intracerebral lactate/pyruvate ratio (44–46) that alter the brain functional organization by modifying neuronal networks connectivity (43, 47, 48). These data suggest that the disruption of attention and working memory systems underlying delirium might be a consequence of a functional breakdown due to diminished connectivity and/or network integration within different brain region (2, 49, 50). In our context, the stress that metabolic changes related to epileptic activities -even sporadic- inflict on older fragilized brain might represent a precipitating factor interfering with brain network functioning and thus triggering or contributing to delirium symptoms. Finally, in favor of a role of epileptic activities on delirium symptomatology, a high percentage of patients receiving AEDs showed clinical improvement (65%). Besides the 6 patients with NCSE and the 4 of the 6 patients treated on clinical bases prior to the beginning of EEG, this concerned two patients with patterns in the continuum ictal/interictal (GPDs and Multifocal discharges), indicating that early treatment with AED might be beneficial even for cases in which EEG is not defined as ictal. However, the potential implication of SEDs on delirium symptomatology should be balanced by the fact that SEDs are common findings in patients over 65 years old. Indeed, SEDs are found in up to 16% of EEG realized for transient alteration of consciousness or focal neurological symptoms (resolved at the time of the recordings) and cognitive impairment in older adults ambulatory clinics (51); and may be found as often as 40% of Alzheimer patients (52). Furthermore, delirium precipitating factors, in our patients were corrected simultaneously with AED administration making the specific contribution of each intervention hard to disentangle. Dedicated investigation on larger groups of patients to focus on the role of AEDs are therefore needed to assess the exact role of SEDs on delirium pathophysiology and the yield of AED in that context.

Taken together, these arguments suggest that SEDs and not only NCSE may play a role in delirium symptoms and pathophysiology. To establish a more definite correlation between delirium symptoms and epileptic activities, dedicated studies controlling EEGs after delirium resolution or improvement and showing that epileptic activities have disappeared would strengthen our hypothesis. Epileptic activities may also be involved in the poor cognitive long-term outcome observed for delirious patients (2, 5, 53): repeated epileptic insults could induce neuronal loss resulting in long-term cognitive deterioration (42, 54, 55) leading to poor performances on global cognitive evaluation when using Addenbrooke's Cognitive Evaluation test in epileptic adults with abundant SEDs (42).

To conclude, this study confirms the association between epileptic activities and acute delirium in older individuals suggesting a pathophysiological relation. In that context, epileptic activities need to be searched for by cEEG irrespectively of the presence of acute medical conditions. Epileptic activities, triggered, or not by acute medical conditions, could lead to brain dysfunction and subsequently explain delirium symptoms in a substantial proportion of patients. Further, studies are needed to clarify the role of AEDs in preventing and treating delirium by investigating more closely EEG modifications after resolution of delirium even without AEDs.

## Impact Statement

1/ We certify that this work is novel and confirmatory

2/ Potential impact of this work involves:

Management of delirium in acute phase and possibly long-term outcome.

## Ethics Statement

The study had prior approval by the Ethics Committee of Erasme Hospital (021/406). Patients were included either after written informed consent or in cases where delirium symptoms prevented written informed consent from the patients, the informed consent was obtained from the closest relative.

## Author Contributions

SS and GN designed the study, analyzed the data, and wrote the manuscript. NG, BL, and CD analyzed the EEG and drafted the manuscript for intellectual content. SD drafted the manuscript for intellectual content.

### Conflict of Interest Statement

The authors declare that the research was conducted in the absence of any commercial or financial relationships that could be construed as a potential conflict of interest.
